# Characteristics of limb kinematics in the gait disorders of post-stroke patients

**DOI:** 10.1038/s41598-024-53616-w

**Published:** 2024-02-07

**Authors:** Naomichi Mizuta, Naruhito Hasui, Takumi Kai, Yasuhiro Inui, Masahiro Sato, Sora Ohnishi, Junji Taguchi, Tomoki Nakatani

**Affiliations:** 1 Department of Rehabilitation, Faculty of Health Sciences, Nihon Fukushi University, 26-2 Higashihaemi-cho, Handa-shi, Aichi 475-0012 Japan; 2 Neurorehabilitation Research Center, Kio University, 4-2-2 Umaminaka, Koryo, Kitakatsuragi-gun, Nara 635-0832 Japan; 3 Department of Therapy, Takarazuka Rehabilitation Hospital, 22-2 Tsurunoso, Takarazuka-shi, Hyogo 665-0833 Japan; 4 Department of Neurorehabilitation, Graduate School of Health Sciences, Kio University, 4-2-2 Umaminaka, Koryo, Kitakatsuragi-gun, Nara 635-0832 Japan; 5 Department of Rehabilitation Medicine, Hatsudai Rehabilitation Hospital, 3-53-3 Honmachi, Shibuya-ku, Tokyo, 151-0071 Japan; 6 Department of Rehabilitation, Nara Prefecture General Rehabilitation Center, 722 Oo, Tawaramoto, Shiki-gun, Nara 636-0393 Japan; 7 Department of Rehabilitation, Nakazuyagi Hospital, 1-31, Nakazu, Tokushima-shi, Tokushima 770-0856 Japan; 8 Department of Medical, Takarazuka Rehabilitation Hospital, 22-2 Tsurunoso, Takarazuka-shi, Hyogo 665-0833 Japan

**Keywords:** Neurological disorders, Diseases of the nervous system, Motor control, Neuronal physiology

## Abstract

Post-stroke gait disorders involve altered lower limb kinematics. Recently, the endpoint of the lower limb has been used as a control variable to understand gait kinematics better. In a cross-sectional study of sixty-seven post-stroke patients, the limb extension angle and effective limb length during gait were used as input variables with a mixed Gaussian model-based probabilistic clustering approach to identify five distinct clusters. Each cluster had unique characteristics related to motor paralysis, spasticity, balance ability, and gait strategy. Cluster 1 exhibited high limb extension angle and length values, indicating increased spasticity. Cluster 2 had moderate extension angles and high limb lengths, indicating increased spasticity and reduced balance ability. Cluster 3 had low limb extension angles and high limb length, indicating reduced balance ability, more severe motor paralysis, and increased spasticity. Cluster 4 demonstrated high extension angles and short limb lengths, with a gait strategy that prioritized stride length in the component of gait speed. Cluster 5 had moderate extension angles and short limb lengths, with a gait strategy that prioritized cadence in the component of gait speed. These findings provide valuable insights into post-stroke gait impairment and can guide the development of personalized and effective rehabilitation strategies.

## Introduction

Gait is a complex motor task that requires coordinated movements of multiple body systems, including the musculoskeletal, neurological, and sensory systems^1^. In post-stroke patients, these systems are often impaired, leading to gait disorders affecting gait independence, fall risk, and quality of life^2–5^. It is important to understand the pathophysiology of gait disorders in post-stroke patients to develop effective rehabilitation strategies.

Factors such as severity of motor paralysis, sensory impairment, spasticity, instability, and gait strategy contribute to gait disorders in post-stroke patients. For example, the severity of motor paralysis and spasticity causes abnormal muscle activity patterns that affect lower limb kinematics during gait^6–9^. Sensory impairment and loss of balance lead to impaired lower limb motor coordination, stride length, and balance^10–12^. Gait strategies, such as cadence to gait speed ratios, alter limb kinematics and compensate for gait disorders by decreasing gait efficiency^13,14^. These factors result in a pattern of joint motion of the paretic leg during gait that deviates from that of a normal individual. Specifically, the altered motion often manifests as an extension thrust or a stiff knee gait pattern^15^. Such abnormal gait patterns are associated with functional impairments like spasticity. In contrast to joint kinematics, which captures individual joint movement characteristics, limb kinematics considers movement characteristics of the entire lower limb, allowing us to understand the coordination and control of multiple segments and to evaluate more global movements^16–18^. Compared to the individual control of each joint, limb kinematics controls the lower limb’s motion during gait with fewer degrees of freedom^17,19,20^. These plane laws are expressed by limb angle and length, which provide the basis for human gait kinematics. The motion of the hip and knee joints can be approximated from the kinematics of the limb kinematics. However, the motion of the ankle joint is difficult to derive from the kinematics of the limb kinematics. Ankle motion can be estimated from limb angle in the stance phase, but neither limb angle nor length reflects ankle motion in the swing phase. It is clinically significant to note that limb kinematics is beneficial because it contributes more than joint kinematics to the recovery of gait speed in post-stroke patients^20^.

However, in clinical rehabilitation, we frequently encounter cases where the relationship between the limb flexion or extension angle and length diverges, such as a combination of low limb extension angle and short limb length or vice versa. Subgroups with such divergent relationships may have different characteristics, such as motor paralysis severity, sensory impairment, spasticity, loss of balance, and gait strategy. A comparison of these factors between subgroups will provide a deeper understanding of the fundamental mechanisms that contribute to gait disorders in post-stroke patients and will contribute to the development of individualized and effective rehabilitation strategies tailored to the specific patterns of each subgroup. However, it remains unclear whether subgroups with different limb kinematic patterns exist in post-stroke patients.

This study examined the relationship between limb angle and length on the paretic side during gait in post-stroke patients. We also identified subgroups in these distributions using mixed Gaussian clustering and examined the characteristics of each cluster and its clinical and gait assessment. We assumed the identified clusters would be characterized based on clinical and gait assessments, such as motor paralysis severity, spasticity, balance ability, and gait strategy. This study is the first to use clustering based on a mixed Gaussian model to subgroup and characterize limb kinematics features during gait in post-stroke patients. The results of this study are expected to provide insights into the heterogeneity of limb kinematics patterns in post-stroke patients and have strong implications for researchers in motor control, neurophysiology, or biomechanics by identifying subgroups with distinct gait characteristics.

## Results

Sixty-seven participants were enrolled in this cross-sectional study. All participants performed a gait task at a comfortable speed, during which videos were recorded from the sagittal plane to calculate the limb kinematics of the paretic leg. The limb kinematics consisted of two variables: limb angle (the peak lower limb extension angle with positive values in the flexion direction) and limb length. The former is the minimum value of the angle between the centers of the hip and ankle joints in the sagittal plane and the vertical axis during gait (i.e., the extension position). The latter is calculated by dividing the distance between the hip and ankle joint centers by the sum of the distances between the hip and knee joint centers and between the knee and ankle joint centers. The minimum value during the swing phase was used as the limb length. Each participant’s different limb angle and length sets were examined using the Gaussian mixture model (GMM)-based probabilistic clustering with limb angle and length as input variables. We also examined different combinations of limb angles and lengths for each participant. Differences in clinical and gait characteristics between the clusters were also identified.

### Gaussian mixture model-based cluster analysis

**Figure 1:**
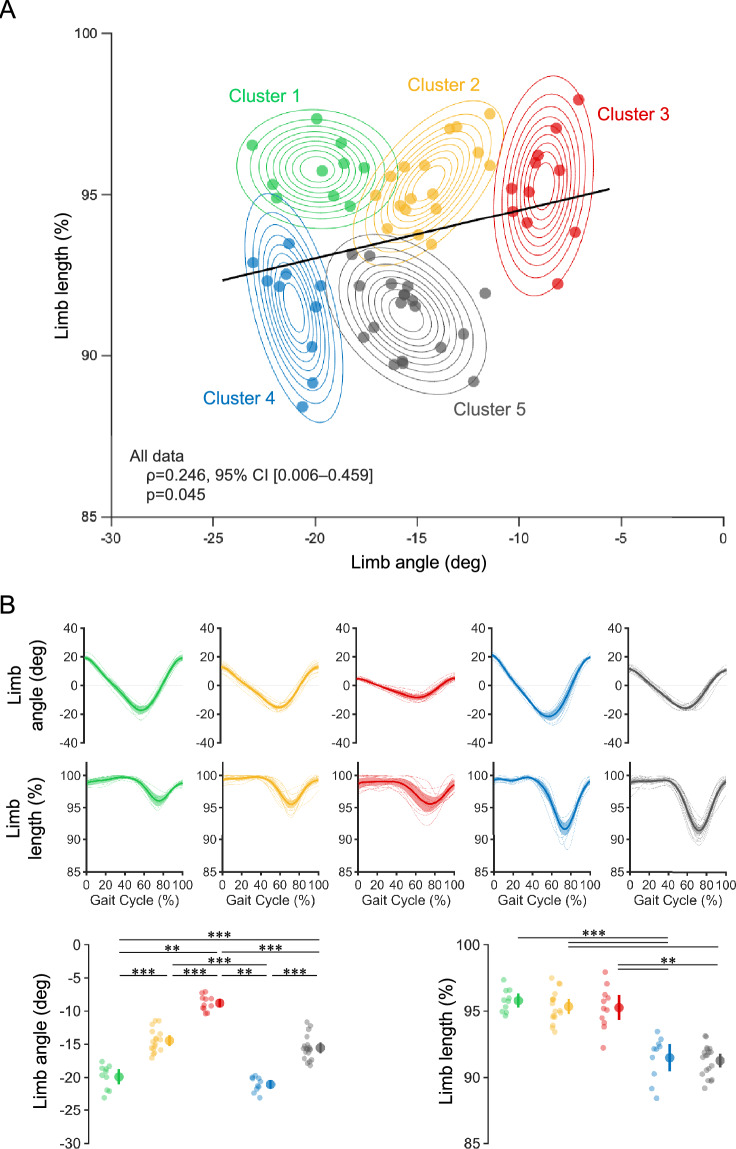
Summary of the Gaussian mixture model-based cluster analysis. (**A**) Scatterplots of each cluster’s limb angle and length based on mixed Gaussian clustering and the outermost 95% confidence ellipsoid. The black line represents the regression line for all data. The limb angle and length scatterplots and 95% confidence ellipses show the independence and characteristics of the five clusters. The direction of flexion in the limb angle was defined as positive. (**B**) Limb angle and length at a comfortable gait speed in each cluster. The top panels represent time-series data, the deep lines represent means, the area plots represent 95% confidence intervals, and the thin lines represent plots of individual participants within a cluster. The bottom row shows the limb angle extension peak and limb length peak values, which are the results of the statistical comparisons of scores among the clusters. The direction of flexion in the limb angle was defined as positive. The plot includes means, 95% confidence intervals, and individual participant data. Asterisks represent statistical significance determined by post-hoc testing (Steel–Dwass test) using the Kruskal–Wallis test. **P < 0.01, ***P < 0.001.

Limb extension angle and length were associated with each other (ρ = 0.246, 95% confidence interval [CI] [0.006–0.459], *P* = 0.045). However, the scatter plots of the limb extension angle and length showed large variances, with low/high values for the limb extension angle and high/low values for the limb length. Therefore, to classify subgroups in the association between limb extension angle and length, we performed GMM-based clustering using these two variables. Among the several models that met these criteria, the model that showed high theoretical validity with better Bayesian information criterion (BIC) and integrated complete data likelihood (ICL) was selected as the optimal model (Table 1). Five clusters were identified from the limb extension angle and length distributions. Figure 1A shows a scatterplot of each cluster’s limb angle and length, with the outermost ellipse representing the 95% confidence level of the distribution, indicating the independence and characteristics of each cluster. Clusters 2, 3, 4, and 5 were depicted as ellipses with a wide range of limb length variance, while the limb angle variance was comparatively small. However, Cluster 1 was represented by an ellipse with a large variance in limb angle. The 95% confidence level perimeter, constructed based on the distribution of each cluster, showed partial overlap in Clusters 1, 2, 4, and 5.

**Table 1:** Criteria for selecting the number of clusters.

Number of clusters	Bayesian information criterion	Integrated complete data likelihood
4	770.0	313.5
5	765.4	311.2
6	771.4	314.2
7	771.4	314.2

This table presents the Bayesian information criterion and the integrated complete data likelihood for the Gaussian mixture model-based probabilistic clustering with 4–7 clusters. Lower values for both indicators indicate a superior model fit. The optimal model performance is observed when five clusters yield the lowest values for BIC and ICL among the considered range.

The limb extension angle differed between clusters (χ^2^ = 54.691, df = 4, ε^2^ = 0.829, *P* < 0.001), with Clusters 1 and 4 significantly higher than Clusters 2 and 5. Limb length also differed between clusters (χ^2^ = 47.947, df = 4, ε^2^ = 0.726, *P* < 0.001), with Clusters 1, 2, and 3 being significantly longer than Clusters 4 and 5 (Fig. 1B). In particular, Cluster 3 appeared to have a large intragroup variance.

### Clinical and gait characteristics

**Figure 2:**
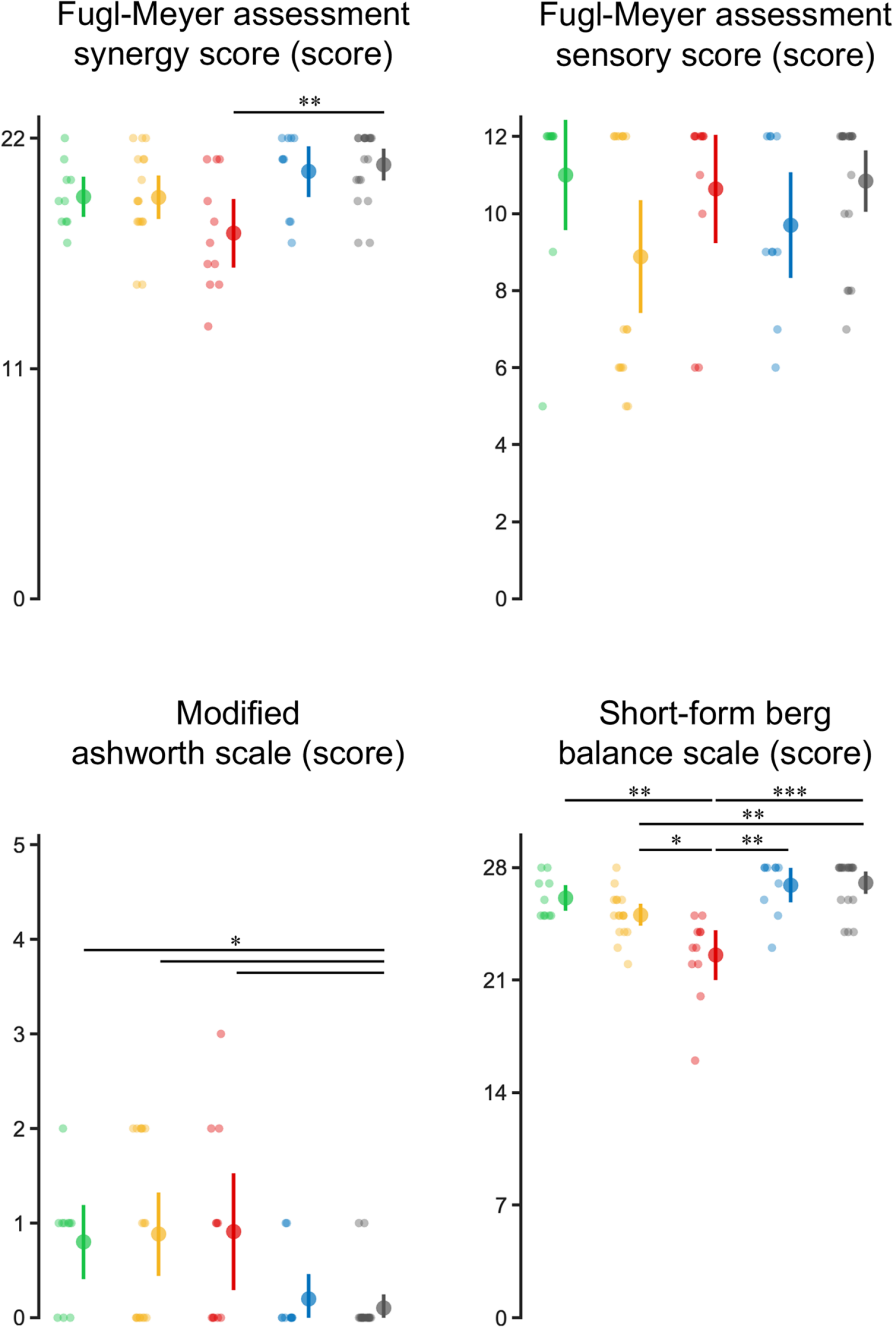
Clinical evaluation scores in each cluster. Each panel shows the results of the statistical comparison of scores across clusters. Clusters 1 to 5 are shown from left to right, corresponding to the colors. Means, 95% confidence intervals, and individual participant data are plotted. Asterisks indicate statistical significance determined by post-hoc testing (Steel–Dwass test) using the Kruskal–Wallis test. *P < 0.05, **P < 0.01, ***P < 0.001.

**Figure 3:**
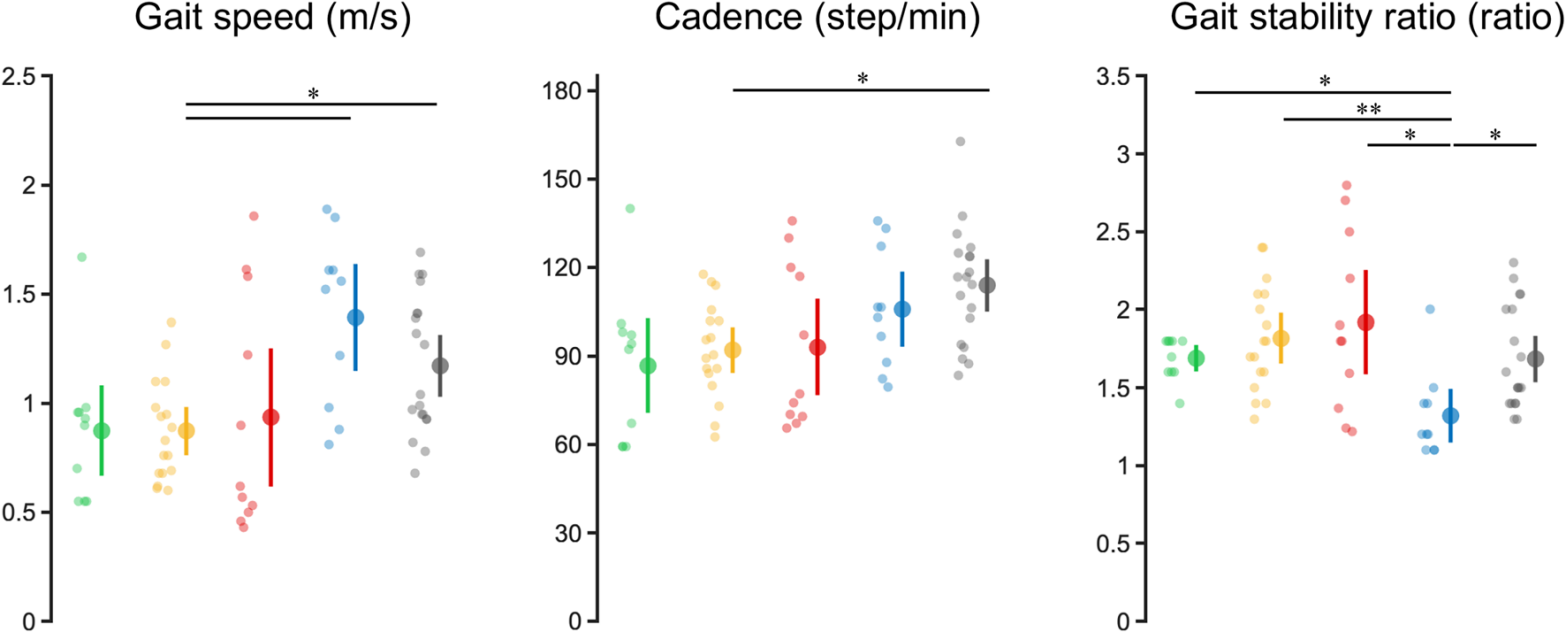
Gait evaluation scores in each cluster. Each panel shows the results of the statistical comparison of scores across clusters. Clusters 1 to 5 are shown from left to right, corresponding to the colors. Means, 95% confidence intervals, and individual participant data are plotted. Asterisks indicate statistical significance determined by post-hoc testing (Steel–Dwass test) using the Kruskal–Wallis test. *P < 0.05, **P < 0.01.

The results of the clinical and gait evaluations for each cluster are summarized in Figs. 2 and 3, respectively, and their demographic characteristics are listed in Table 2. Each cluster yielded different results for each evaluation. In the clinical evaluation, the Fugl–Meyer assessment Synergy (FMS) score was lower in Cluster 1 than in other clusters. However, the Fugl–Meyer Assessment (FMA) sensory score did not differ among the clusters. The plantar flexors’ modified Ashworth Scale (MAS) score was higher in Clusters 1, 2, and 3 than in Cluster 5. The patients on the right side of the scattergram in Clusters 2 and 3 had lower scores than the others in the Short-Form Berg Balance Scale, indicating lower balance ability, except for Cluster 5, in the limb extension angle and length distribution. Gait speed was higher in Clusters 4 and 5 than in Cluster 2. Cadence was higher in Cluster 5 than in Cluster 2, and the gait stability ratio was lower in Cluster 4 than in the other clusters, indicating that cadence to gait speed ratio was small (i.e., a strategy favoring stride extension). Based on these results, Cluster 1 was characterized by a high limb extension angle and limb length and high MAS values of the plantar flexors, which can be attributed to increased muscle tone. Cluster 2 had a moderate limb extension angle and high limb length and was characterized by increased muscle tone and decreased balance ability. Cluster 3 was characterized by low limb angle and high limb length, increased muscle tone, decreased balance ability, and severe motor paralysis. Cluster 4 had a high limb extension angle and short limb length and was milder in all clinical evaluations than the other clusters. Finally, Cluster 5 had a moderate limb extension angle and short limb length and was mildly affected in all clinical evaluations.

**Table 2:** Characteristics of each cluster.

	Cluster 1	Cluster 2	Cluster 3	Cluster 4	Cluster 5	All participants
Age (years)	63.4 ± 11.1	63.9 ± 11.0	56.5 ± 21.3	55.7 ± 11.5	62.8 ± 17.6	61.1 ± 15.1
Time since stroke (days)	85.9 ± 16.4	76.2 ± 21	98.0 ± 33.9	65.3 ± 20.9	72.6 ± 20.8	77.6 ± 23.4
Sex (n): male/female	8/2	12/5	6/5	5/5	11/8	42/25
Affect side (n): right/left	9/1	9/8	4/7	7/3	6/13	35/32
Functional ambulation category	4.90 ± 0.32	4.17 ± 0.64	4.18 ± 0.75	4.50 ± 0.71	4.63 ± 0.68	4.46 ± 0.68
Using assist device (n): no use/T-cane/Q-cane	1/9/0	0/17/0	4/6/1	7/3/0	14/5/0	26/40/1

The data are reported as means or n ± standard deviations.

Interestingly, Clusters 4 and 5, which had the fastest gait speeds, showed no differences in gait speed, FMS score, FMA sensory score, spasticity score, and balance ability. However, the gait stability ratio was lower in Cluster 4, indicating that Cluster 4 prioritized stride length and Cluster 5 prioritized cadence in their strategies for configuring gait speed. The detailed statistical results are presented in Table 3 (post hoc).

**Table 3:** Comparison of clinical and gait evaluations among each cluster.

Steel–Dwass	Fugl–Meyer assessment Synergy score	Fugl–Meyer assessment Sensory score	Modified Ashworth scale	Short–form Berg balance scale	Gait Speed	Cadence	Gait stability ratio
Cluster	*P* value	*P* value	*P* value	*P* value	*P* value	*P* value	*P* value
1 versus 2	1	0.471	1	0.451	0.999	0.944	0.927
1 versus 3	0.544	0.972	1	0.003	0.984	0.955	0.934
1 versus 4	0.585	0.530	0.166	0.557	0.119	0.455	0.030
1 versus 5	0.137	0.950	0.010	0.311	0.215	0.095	0.963
2 versus 3	0.454	0.746	1	0.021	0.955	1	0.999
2 versus 4	0.685	0.940	0.297	0.059	0.017	0.493	0.008
2 versus 5	0.140	0.290	0.030	0.007	0.035	0.015	0.726
3 versus 4	0.074	0.822	0.380	0.005	0.356	0.667	0.046
3 versus 5	0.009	1	0.051	< 0.001	0.461	0.332	0.910
4 versus 5	0.988	0.669	0.959	0.997	0.568	0.923	0.022

This table shows the *P* values determined by the post-hoc tests (Steel–Dwass) for each cluster’s clinical and gait evaluations.

## Discussion

This study investigated the association between limb angle and length during gait in post-stroke patients and identified clusters based on probabilistic clustering. Limb extension angle and length were positively correlated but deviated from this correlation in some participants. The clustering of participants based on limb angle extension and length identified five clusters with different combinations of these variables, each exhibiting unique clinical and gait characteristics. Each cluster provides a useful framework for characterizing such deviations and may serve as a basis for developing tailor-made intervention strategies for post-stroke patients, along with the need to decompose limb kinematics into two components for observation.

### Association between limb angle and length during gait

Our findings provide new insights into limb kinematics during gait in post-stroke patients. Clustering based on a Gaussian mixed model with limb extension angle and length as input variables identified five clusters with different associations between limb extension angle and length. Gaussian mixed model-based clustering is a probabilistic model-based clustering method that is more robust than other clustering methods^21,22^. In a previous study that classified the features of joint kinematics during gait in post-stroke patients, the excessive knee extension pattern group was considered a group similar to Clusters 1, 2, and 3, with high values of limb length in this study^5^. However, previous studies did not record hip kinematic data, thus making it difficult to infer a relationship with limb angle in this study. Furthermore, previous studies have not assessed spasticity or balance ability, and we believe it is significant to identify subgroups of gait patterns, including dysfunctional characteristics, as we did in this study. Although a previous longitudinal study investigated limb kinematics during gait in post-stroke patients^20^, no study has identified subgroups of limb kinematics. These clusters are associated with different clinical and gait characteristics, highlighting the importance of considering individual differences in limb kinematics when evaluating the gait of post-stroke patients. Importantly, although our findings showed a positive association between limb extension angle and length, the distribution of these variables varied (Fig. 1A). This is because some participants indicated low values for one variable and high values for the other. Clustering participants based on the joint distribution of these two variables allowed us to identify subgroups of participants with different combinations of limb extension angles and lengths and to gain a more sensitive understanding of the association between these variables. Previous studies have often covered limb kinematics variables inclusively^14,20,23^. As shown in the scatterplots of limb extension angle and length, they were positively correlated, but their variance was large. Moreover, participants with different combinations of these variables may have different pathophysiological characteristics underlying gait disturbance (Fig. 1A). Conversely, the 95% confidence level perimeter, constructed based on the distribution of each cluster, partially overlapped in Clusters 1, 2, 4, and 5. These overlapping regions may contain clusters with latent characteristics. In this study, the optimal model for determining the number of clusters was selected based on BIC and ICL. Nevertheless, it is possible that increasing the sample size could potentially unveil new clusters.

### Clinical and gait characteristics for each cluster

Our results demonstrate that different clusters of limb kinematics are associated with different clinical and gait characteristics (Figs. 2, 3). Cluster 1 was characterized by increased muscle tone of the plantar flexors that may reflect spasticity, as evidenced by the higher limb extension angle, higher limb length, and higher MAS score. This cluster also had a higher balance ability, as indicated by a higher Short-Form Berg Balance Scale score. Cluster 2 was characterized by moderate limb extension angle, high limb length, increased muscle tone, and decreased balance ability. Previous studies have shown that decreased balance ability is associated with decreased stride length^11,24,25^. Cluster 2 differed from Cluster 1 in that it had a lower score on the Short-Form Berg Balance Scale, showing that an increase in the limb angle was a strategy to avoid compromising stability during gait. This cluster is considered a transitional state between Clusters 1 and 3. Cluster 3 was characterized by a low limb extension angle and high limb length, along with increased muscle tone, further loss of balance ability, and more severe motor paralysis. The severity of the motor paralysis is related to the propulsive force of the paretic leg^7^, and the trailing limb angle is important in producing this force^26^. The trailing limb angle is a measurement similar to the lower limb extension angle, with the distal part of the lower limb vector represented by the fifth metatarsal head or the center of the ankle joint. Increasing the lower limb extension angle requires notable force from the plantar flexor muscles of the ankle joint. However, because of the effects of motor paralysis, this force is considered insufficient. Cluster 3 differed from Cluster 2 in that its Short-Form Berg Balance Scale score was even lower than that of Cluster 2, demonstrating that stability during gait is severely impaired. The graded differences in limb extension angle and Short-Form Berg Balance Scale score between Clusters 1, 2, and 3 indicate associate between decreased gait stability and reduced limb extension angle (Figs. 2, 3).

Our results show that Clusters 1, 2, and 3, which are located at the top of the scattergram in Fig. 1A, have a common clinical characteristic of having a higher MAS score of the plantar flexors than Clusters 4 and 5 and that a higher MAS score of the plantar flexors is associated with a higher limb length, which may be characterized by an unable to shorten the lower limbs during gait because of spasticity. The severity of spasticity affects the decrease in lower limb joint motion during gait^6^. Cluster 4 had a high limb extension angle and short limb length and showed mild illness results in all clinical evaluations compared with the other clusters. Interestingly, this cluster had the lowest gait stability ratio, indicating a preference for stride length in the gait strategy. Finally, Cluster 5 had a moderate limb extension angle and short limb length, with mild results in all clinical evaluations. Clusters 4 and 5 participants had the fastest gait speeds and mildest severity of motor paralysis, whereas Cluster 4 had the lowest gait stability ratio and Cluster 5 had the highest. Furthermore, Clusters 4 and 5 did not differ in limb length, indicating that the stance phase factor (i.e., limb extension angle) was relevant to the composition of gait speed in these clusters. Since this study only measured a single time point, it remains uncertain whether the gait stability ratios in Clusters 4 and 5 are outcomes of recovery or learning effects following the onset of stroke. Nevertheless, as strategies for constructing gait speed, Cluster 4 prioritizes stride length, and Cluster 5 prioritizes cadence (Fig. 3). These results demonstrate that patients use different gait strategies to achieve similar gait speeds, which may have implications for developing more efficient and effective rehabilitation strategies.

This study is the first to classify and characterize subgroups of limb kinematics during gait in post-stroke patients using clustering. The limb kinematics subgroups identified in this study are characterized by unique patterns and combinations of motor paralysis, spasticity, balance ability, and gait strategies in each cluster. It is anticipated that interventions such as therapeutic electrical stimulation, transcranial direct current stimulation, and gait robots based on these cluster characteristics can contribute to developing personalized and effective rehabilitation strategies.

### Limitations and future direction

This study has some limitations that should be considered when interpreting the results. First, the cross-sectional design did not allow for causal inferences regarding the association among limb kinematics, clinical characteristics, and gait ability. Future longitudinal studies are required to investigate the effects of interventions based on the identified clusters. Second, the relatively small sample size may limit the generalizability of the results. Future studies should examine causal relationships in larger samples using more comprehensive measures. Third, motor paralysis, spasticity, and balance ability, which characterize each cluster, were not quantified during gait. Given the discrepancy between dysfunction and performance, it is necessary to obtain these variables during gait using electromyography and motion analysis devices. Fourth, OpenPose was used to identify limb kinematics, a potential limitation since no previous studies have validated the accuracy of this software for gait motion in post-stroke patients. Finally, because we only investigated limb kinematics during gait at a comfortable speed, we could not determine the whole range of motor control mechanisms in post-stroke patients. Other gait conditions need to be assessed to analyze the pathophysiology of gait disturbance in detail because gait at a comfortable speed does not allow us to distinguish between pure disability or compensatory strategies post-stroke^8,23,27^. The clustering analysis used only two variables (limb extension angle and length) to classify participants into clusters. Although these variables are important in determining lower limb kinematics during gait, other variables, such as joint angle in the frontal and horizontal plan, joint moment, muscle activation, and even trunk function, may have contributed to the clustering of participants.

Furthermore, a distinction in the paretic side can be observed between Clusters 1 and 5 (Table 2). Given that the location of the lesion is associated with post-stroke gait disorders and that corticospinal tract injury affects the kinematic pattern^28–30^, we believe that future studies using voxel-based lesion-symptom mapping and diffusion tensor tractography will provide a more comprehensive understanding of the pathophysiology. Future studies should investigate participant clustering using a more comprehensive set of variables better to understand the association between limb kinematics and clinical characteristics.

## Materials and methods

### Participants

In this cross-sectional study, Sixty-seven post-stroke individuals (mean ± standard deviation, 61.1 ± 15.1 years; stroke onset, 77.6 ± 23.4 days) were enrolled at the Takarazuka Rehabilitation Hospital, Hatsudai Rehabilitation Hospital, Nara Prefecture General Support Center for Persons with Disabilities, and Nakazuyagi Hospital. This study used the Strengthening the Reporting of Observational Studies in Epidemiology checklist. The exclusion criteria were as follows: (1) inability to walk independently without the assistance of physical therapists, (2) inability to walk without using leg braces, (3) presence of bilateral lesions, (4) Mini-Mental State Examination score < 24 points, (5) a history of orthopedic disease, (6) presence of pain, (7) presence of cerebellar lesions or resting tremor, and (8) presence of unilateral spatial neglect, except in the context of stroke. Patients who did not meet any of the exclusion criteria were enrolled. All participants provided informed consent prior to the start of this study. All procedures were approved by the Ethics Committee of Takarazuka Rehabilitation Hospital (ethics review number: 20200011) and conducted according to the Declaration of Helsinki.

### Experimental setup and procedures

The participants were instructed to walk thrice on a 10-m walkway with a supplementary 6-m walkway, with a physical therapist standing near them to eliminate the risk of falling. The participants were allowed to use a cane as necessary during the assessments; however, lower-limb orthotics were not allowed. During gait, video recording was performed in the sagittal plane (sampling rate, 120 Hz). Before recording, we confirmed a score of < 4 on the modified Borg scale to ensure that fatigue did not affect performance.

### Clinical evaluation

The FMA was used to measure the severity of lower limb motor paralysis and sensory disturbance. The FMS score was used to determine the FMA motor score^31^. We assessed the ankle plantar flexor muscle responses using a MAS converted to a 0–5-point scale (0, no increase in muscle tone; 5, the affected part is rigid in flexion or extension) to evaluate spasticity. We also assessed motor performance using the short-form Berg Balance Scale and Functional Ambulation Category.

### Data recording and analysis

**Figure 4:**
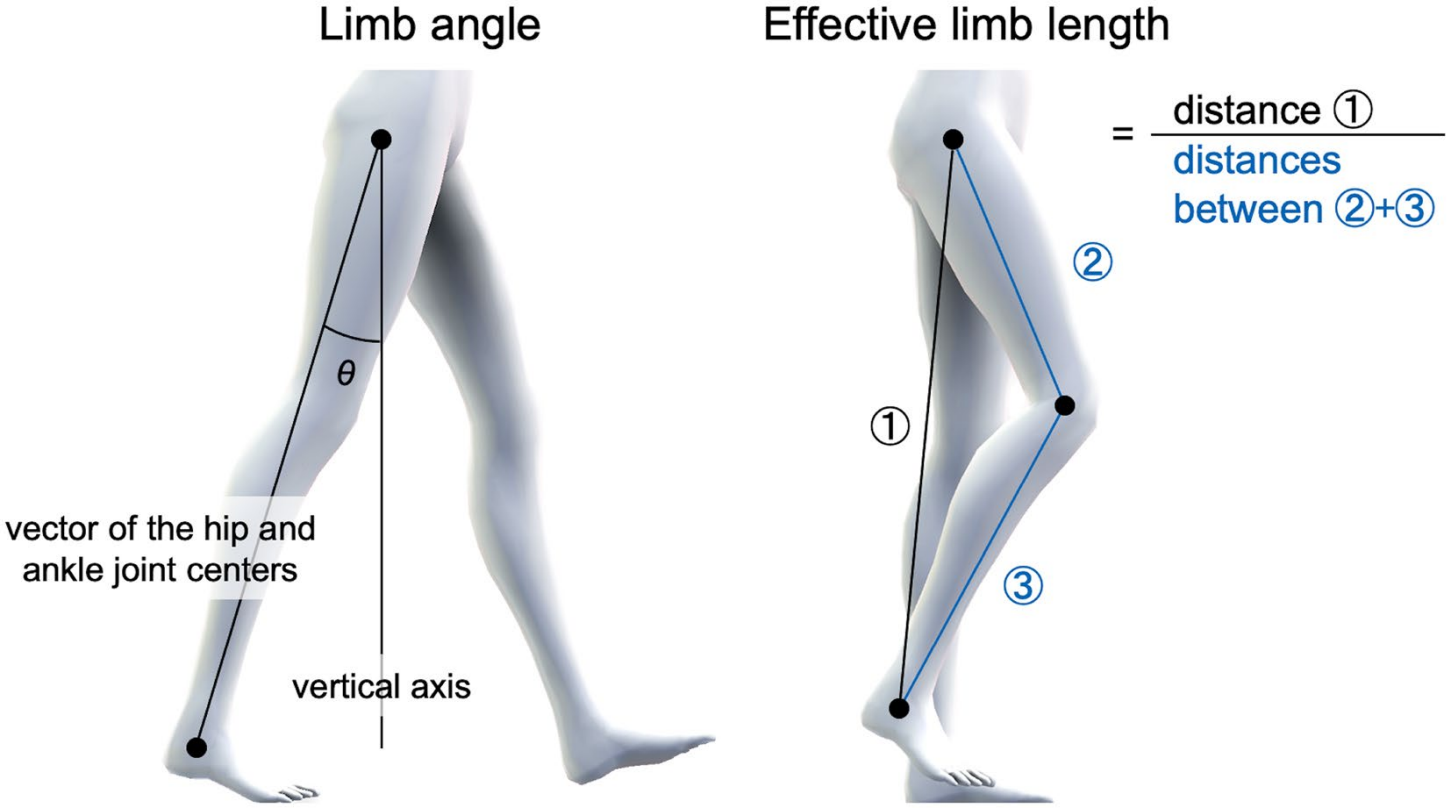
Calculation method of limb kinematics. This figure illustrates the method used to calculate limb kinematics. The limb angle was determined as the angle between the vertical axis and the vector connecting the hip and ankle joint centers, with the peak value in the extension direction defined as the limb angle. The distance between the hip and ankle joint centers was divided by the sum of the distances between the hip and knee joint centers and between the knee and ankle joint centers to calculate limb length. The effective limb length was defined as the minimum value observed during the swing phase.

The participants walked at a comfortable speed. The participants practiced gait at a comfortable speed for 5 min to familiarize themselves with the procedure before data collection. Using the recorded video data, gait speed, and cadence were measured using a stopwatch when the participants walked between the start and end lines of the 10-m walkway^32^. The first and last three gait cycles were removed from the dataset to prevent any confounding effects because of acceleration and deceleration. Ten strides were then extracted from each gait session. The coordinates of the hip and ankle joint centers were obtained using OpenPose (version 1.6.0, https://github.com/CMU-Perceptual-Computing-Lab/openpose) in the recorded video analysis to calculate the joint angle^33^. OpenPose is a markerless motion capture system that estimates posture from video camera images, and its reliability has been confirmed by optical motion capture^34–36^. The angle data were low-pass filtered with a cut-off of 6 Hz using a zero-lag fourth-order Butterworth filter^37^. The limb angle was calculated as the angle between the vertical axis and vector of the hip and ankle joint centers^38^, and the peak value in the extension direction was defined as the limb angle^20,39^. The direction of flexion was defined as positive. Limb length was calculated by dividing the distance between the hip and ankle joint centers by the sum of the distances between the hip and knee joint centers and between the knee and ankle joint centers (Fig. 4)^20,40^. The minimum value during the swing phase was used as the effective limb length. Limb angle and length were measured on the paretic side and were averaged over 10 gait cycles. Time-series data on the shank angle were differentiated once to convert them into angular velocity data, after which the heel contact time on the paretic side was determined to identify gait events from joint angles. Each gait cycle was spline interpolated to normalized times on a 201-point gait cycle time basis.

### Gaussian mixture model clustering for limb kinematics characteristics

Limb extension angle and length data were used to classify participants using GMM-based clustering; GMM clustering is a probabilistic model-based clustering method and is more robust than other clustering methods^21^. In this clustering method, differences in the number of clusters, distribution, volume, shape, and orientation can be compared using statistical information criteria, such as BIC, ICL, or both criteria^22^. Two criteria were defined for the number of clusters and distribution features: (1) the number of clusters was between four and seven (aiming for a more detailed classification than the four categories of high/low limb extension angle and high/low limb length), and (2) the model distribution parameters were not equally distributed. Among several models meeting these conditions, the model with good BIC and ICL values and high theoretical validity was selected as the optimal model. MATLAB R2019b (MathWorks, Inc., Natick, MA, USA) was used for all the data analyses.

### Statistical analyses

Spearman’s rank correlation coefficient was used to confirm the distribution of limb extension angle and length. Differences in basic attributes, clinical test scores, and gait parameters in each cluster were examined using the chi-squared and Kruskal–Wallis tests (post hoc Steel–Dwass test). Statistical significance was set at *P* < 0.05. All statistical analyses were performed using R version 4.1.0.

## Data Availability

The data used in this study are available upon request in an anonymized format from the corresponding author.
